# Clinical comprehensive of microfracture, autologous chondrocyte implantation, and periosteum-covered iliac bone grafting for Hepple stage IV–V lesions

**DOI:** 10.1097/MD.0000000000039586

**Published:** 2024-09-20

**Authors:** Heda Liu, Lin Liu, Ming Li, Fei Huang, Pengwei Li, Sheng Liu

**Affiliations:** a Department of Foot and Ankle Surgery, Cangzhou Traditional Chinese and Western Medicine Combined Hospital, Cangzhou, Hebei Province, China.

**Keywords:** ankle pain, arthroscopy, autologous osteochondral transplantation, Hepple staging, osteochondral lesions of the talus

## Abstract

Chronic ankle pain significantly impairs daily activities and athletic performance with osteochondral lesions of the talus (OLT) in Hepple stages IV and V, which are often causative factors. This study aimed to assess the efficacy and safety of autologous osteochondral transplantation (AOT) for the treatment of these conditions. This retrospective study was conducted from May 2020 to May 2023 at Cangzhou Traditional Chinese and Western Medicine Combined Hospital, including patients with a diagnosis of Hepple stage IV or V OLT confirmed by magnetic resonance imaging (MRI) and arthroscopy. Surgical interventions involved arthroscopic debridement, followed by AOT or limited arthrotomy based on the location and size of the lesion. Preoperative and postoperative evaluations used the Visual Analog Scale, American Orthopedic Foot and Ankle Society Ankle-Hindfoot Scale, MRI-Based Cartilage Repair Tissue Scoring, and the International Knee Documentation Committee Knee Evaluation Form. Statistical analysis was conducted using paired-sample *t* tests to compare the preoperative and postoperative data. Twenty patients were included, revealing significant postoperative improvements in Visual Analog Scale, American Orthopedic Foot and Ankle Society, and MRI-based cartilage repair tissue scores (*P* < .05). The radiographic findings suggested effective cartilage regeneration. No adverse effects were observed in the donor knee sites, as confirmed by the stable pre- and postoperative International Knee Documentation Committee Knee Evaluation Form scores. Recovery of physical abilities was achieved on average within 7.3 weeks for daily activities and 13.4 weeks for sports activities. AOT effectively treats Hepple stage IV–V OLT, improves ankle function, promotes cartilage regrowth, and allows quick resumption of daily and athletic activities without compromising donor-site integrity.

## 1. Introduction

Osteochondral lesions of the talus (OLT) constitute a common clinical condition that significantly affects a broad spectrum of individuals across various age groups. These lesions are frequently manifested as chronic pain upon weight-bearing, reduced joint mobility, ankle swelling, and deterioration in the overall quality of life for the afflicted individuals.^[1,2]^ Trauma is a predominant causative factor for OLT, with nearly half of all patients experiencing acute ankle injuries eventually developing various extents of OLT. Traditionally, arthroscopic debridement, subchondral drilling, and microfracture surgery have been the cornerstone modalities for OLT management.^[3,4]^ Despite their minimal invasiveness and procedural simplicity, these techniques have limited long-term efficacy. One primary drawback is that the resultant repair tissue predominantly consists of fibrocartilage, which lacks the durability and wear resistance of the native hyaline cartilage. Furthermore, in cases with osteochondral separation, specifically those classified as Hepple Stage IV and those with subchondral bone cysts (Hepple Stage V), these traditional approaches have yielded suboptimal outcomes in terms of postoperative cartilage coverage, often leaving patients with persistent chronic ankle pain.^[5,6]^

Emerging therapeutic strategies, such as autologous chondrocyte implantation (ACI) and matrix-induced autologous chondrocyte implantation, offer promise in addressing the limitations of existing surgical methods. These techniques have the advantage of treating lesions irrespective of their size and without donor-site morbidity. Nevertheless, they are hindered by their technical complexity, high associated costs, and necessity for 2-stage surgical procedures, thereby limiting their widespread clinical application.^[7,8]^ Autologous osteochondral transplantation (AOT) is a well-established treatment for knee osteochondral lesions, particularly in Western countries. Although AOT for treating ankle osteochondral lesions is an emerging area of research, there remains a paucity of medium-to-long-term therapeutic efficacy studies conducted domestically.^[9,10]^ This study aimed to fill this gap by evaluating the efficacy of AOT in treating Hepple stage IV–V OLT.

The primary objective of this study is to conduct a comparative clinical evaluation of 3 surgical interventions—microfracture surgery, ACI, and periosteum-covered iliac bone grafting—in the treatment of Hepple Stage V talar osteochondral lesions. Through this comprehensive investigation, we aimed to identify the most effective surgical treatment modality, thereby contributing to the optimization of clinical practice guidelines for the management of advanced-stage OLT.

## 2. Materials and methods

### 2.1. Eligibility and exclusion criteria

This retrospective study was conducted from May 2020 to May 2023 at Cangzhou Traditional Chinese and Western Medicine Combined Hospital. The study specifically sought participants with chronic ankle pain that considerably impeded their daily activities or athletic performance. Eligibility was further narrowed to patients with a diagnosis of either Hepple stage IV OLT or subchondral bone cysts in Hepple stage V OLT, verified through magnetic resonance imaging (MRI) and arthroscopy. The lesion diameters in the enrolled patients had to fall within the range of 0.5 to 2.0 cm. Candidates were excluded from participation if they were diagnosed with Hepple stage I to III OLT or exhibited other disqualifying conditions such as ankle deformity, severe osteoarthritis, poor limb alignment, ankle infection, tumors, or other non-traumatic conditions, as well as any severe systemic or localized diseases. Preoperative evaluations were performed using the Visual Analog Scale (VAS), American Orthopedic Foot and Ankle Society (AOFAS) Ankle-Hindfoot Scale, MRI-Based Cartilage Repair Tissue Scoring (MOCART), and the International Knee Documentation Committee Knee Evaluation Form (IKDC). All participants provided informed consent, and the study protocol was approved by the Ethics Committee of the Cangzhou Traditional Chinese and Western Medicine Combined Hospital.

### 2.2. Arthroscopic exploration and debridement

All surgical procedures were conducted by a single highly experienced orthopedic surgeon specializing in sports medicine. The patient was positioned supine and preoperative markings were performed. Standard sterilization procedures were followed, and a tourniquet was applied for hemostasis. A 4 mm, 30° arthroscope (Stryker Corporation, Kalamazoo, MI) was used for diagnostic and therapeutic ankle arthroscopy. The medial and lateral anterior portals were established for optimal visualization and access to the ankle joint. The arthroscope was introduced, starting at the medial gutter and progressing to the talus dome, trifurcation point, and finally to the lateral gutter. Sites of synovial hypertrophy, ligamentous scarring, and osteophytic impingement were carefully identified and debrided, if present. Meticulous inspection of the central, medial, lateral, and superior regions of the talus, as well as the cartilage beneath the tibia, was performed to assess any chondral defects. The precise locations and dimensions of cartilage damage were documented. Specialized arthroscopic instruments such as curettes, microdrills, and burrs (Stryker Corporation) were employed to perform chondroplasty and drilling of the defect, clean the area down to the subchondral bone to ensure a flat base for potential regenerative procedures, and achieving a 90° angle with the surrounding cartilage walls.

### 2.3. Autologous osteochondral transplantation

For smaller defects located on the lateral or anteromedial aspect of the talar dome, isolated AOT VAS was performed. However, for defects situated on the posteromedial talus or those with a diameter ≥1.2 cm, limited arthrotomy was performed under arthroscopic guidance. In this study, 12 patients underwent isolated arthroscopic transplantation, whereas 8 required additional arthrotomy. During isolated arthroscopic transplantation, a custom-designed ankle distraction device was used to widen the tibitalar joint space while maintaining the ankle in a dorsiflexed position. When arthrotomy was indicated, medial defects were created using an anteromedial ankle incision.

Appropriate diameter instrumentation from an AOT system (Stryker Corporation) was used to drill vertically at the defect site. Generally, a depth of 10 mm is achieved, extending to 15 mm if preoperative MRI indicates a deep subchondral cyst. Osteochondral grafts were harvested from the non-weight-bearing areas of the lateral femoral condyle, matched in size and orientation to the recipient sites, and fixed using a press-fit technique to ensure initial stability. Subsequently, grafts were carefully implanted into the talus to align the transplanted cartilage flush with the surrounding native cartilage to form a contiguous, smooth arcuate surface, thus minimizing the risk of postoperative traumatic arthritis. The defect was either filled with a single graft column or dual-column mosaicplasty was performed if necessary. In either case, no more than 2 columns are used. The extracted bone material was used to backfill the donor sites, followed by hemostasis using a biological adhesive. Finally, the osteotomy cuts were repositioned and secured with 1 to 2 cannulated screws.

### 2.4. Postoperative management and rehabilitation protocol

Upon completion of the surgical procedure, symptomatic treatment is administered to reduce edema, alleviate pain, and sustain elevation of the affected limb. Early rehabilitation commenced with isometric contractions of the lower limb muscles. Starting on the third postoperative day, continuous passive motion exercises of the ankle joint were initiated using a device from Zhejiang Kehui Medical Instruments Co., Ltd. The frequency and duration of continuous passive motion exercises are gradually escalated based on patient recovery up to the maximum achievable range of motion. A protective rehabilitation regime was adopted for the initial 6 weeks. The patients were fitted with an ankle brace and guided through both passive and active ankle joint exercises. Progressive weight-bearing exercises were introduced, ranging from 50% to 75%, and finally to full weight-bearing exercises. Concurrently, strength training for the periankle muscles was incorporated into the regimen.

### 2.5. Justification for sample size and statistical analysis

Prior to conducting the study, a power analysis was performed to determine the appropriate sample size needed to detect a statistically significant difference in the primary outcome measures (VAS, AOFAS, and MOCART scores). Assuming a standard deviation based on preliminary data from similar studies, and aiming for a power of 80% with an *α* level of 0.05, it was calculated that a minimum of 18 patients would be necessary to detect a clinically meaningful difference of 20% improvement in the scores. Considering potential drop-outs and incomplete data, the sample was set at 20 patients. This sample size ensures sufficient power to validate the statistical significance of the observed outcomes, thereby supporting the reliability of our study conclusions. For the purpose of this study, data analysis was performed using IBM SPSS version 27.0. Quantitative metrics, including VAS, AOFAS, MOCART, and IKDC scores, were initially subjected to normality testing using the *F* test. All datasets conformed to a normal distribution and demonstrated homogeneity of variance. Descriptive statistics were presented as mean ± standard deviation. Preoperative and last follow-up quantitative data were compared to determine the effectiveness of surgical interventions. A paired-sample *t* test was used for this purpose. Statistical significance was considered at a *P* value less than .05, indicating meaningful differences between the preoperative and postoperative evaluations.

## 3. Results

### 3.1. Participant analysis

The study included a total of 20 patients: 13 males and 7 females, with ages ranging from 20 to 39 years. All patients presented with chronic ankle pain, ranging in duration from 3 to 39 months, with an average duration of 16 months. All patients underwent clinical examination, radiography, and MRI. Thirteen patients were diagnosed with osteochondritis dissecans (OCD), and 7 had post-traumatic cartilage defects. The lesions were categorized according to the Hepple grading system: 8 cases were type IV and 12 were type V (Table 1).

**Table 1:** Clinical and demographic characteristics of 20 patients with Hepple stage IV and V osteochondral lesions of the talus.

Case number	Age (yr)	Gender	Hepple injury stage	Lesion diameter (cm)	Lesion location	Underlying condition
1	29	Male	V	1.0	Left internal	OCD
2	35	Male	V	0.9	Right external	OCD
3	22	Male	V	1.6	Left internal	OCD
4	27	Male	IV	0.7	Right internal	OCD
5	31	Male	V	2.0	Left external	OCD
6	33	Male	V	0.5	Right internal	OCD
7	28	Male	IV	1.5	Left internal	Post-traumatic
8	25	Male	V	0.9	Left medial external	OCD
9	24	Female	V	1.4	Right external	OCD
10	30	Female	V	1.0	Left internal	Post-traumatic
11	36	Female	V	0.6	Right internal	OCD
12	38	Female	V	1.5	Left internal	OCD
13	26	Male	IV	1.1	Left internal	OCD
14	21	Male	IV	1.3	Right internal	Post-traumatic
15	37	Male	IV	1.8	Right external	Post-traumatic
16	32	Male	IV	1.2	Left internal	Post-traumatic
17	39	Male	IV	1.0	Right external	Post-traumatic
18	20	Female	IV	1.4	Left internal	Post-traumatic
19	23	Female	V	1.3	Right internal	OCD
20	34	Female	V	1.7	Left external	OCD

OCD = osteochondritis dissecans.

### 3.2. Comparison of ankle joint pain, function, and radiographic scores before and after surgery

Substantial improvements were observed in the VAS, AOFAS, and MOCART scores at the last follow-up compared to the preoperative evaluations. The differences were found to be statistically significant (*P* < .05), substantiating the efficacy of surgical intervention (Table 2). Postoperative MRI assessments indicated varying degrees of cartilage coverage and signal intensities consistent with those of the adjacent healthy cartilage. Specifically, hypertrophic cartilage fully covered the defect area in 5 cases, whereas complete coverage with normal cartilage thickness was observed in 12 cases. Partial coverage was noted in 3 cases. In terms of cartilage signal intensity compared to adjacent normal cartilage, 7 cases exhibited consistent signal intensities, while 13 cases showed near-consistent intensities. Postoperative radiographic findings strongly suggest that the surgical technique employed is effective in facilitating cartilage regeneration to varying extents. These promising outcomes, validated by significant improvements in both functional and radiographic scores, reflect the potential of surgical intervention in the targeted patient population.

**Table 2:** Comparative analysis of ankle and knee joint scores pre- and post-intervention in 20 patients.

Parameters	Pre-intervention	Post-intervention	*t* value	*P* value
VAS Score	7.2 ± 1.2	0.8 ± 0.7	19.439	<.001
AOFAS Score	65.1 ± 6.8	94.8 ± 3.1	−12.050	<.001
MOCART Score	3.0 ± 1.0	6.3 ± 1.0	−12.412	<.001
IKDC Score	98.7 ± 0.8	98.1 ± 0.8	0.622	.546

AOFAS = American Orthopedic Foot and Ankle Society, IKDC = International Knee Documentation Committee, MOCART = MRI-based Cartilage Repair Tissue Scoring, VAS = Visual Analog Scale.

### 3.3. Comparative evaluation of ipsilateral knee function before and after surgery

No statistically significant differences were observed in the IKDC scores of the ipsilateral knee joint before and after surgery (*P* > .05, Table 2). This result strongly indicates that the surgical procedure did not compromise knee functionality. Among the participants, 2 patients reported postoperative symptoms of knee pain in the donor area. The pain was managed successfully through conservative treatment and dissipated within a timeframe of 3 to 9 weeks, with an average of 5.6 weeks. No other donor-site complications such as swelling, restricted motion, or fractures were reported. These findings corroborate the safety of the surgical technique used, especially in terms of donor-area morbidity. Moreover, the average time for pain resolution of 5.6 weeks suggests that any postoperative discomfort is likely to be transient and manageable through conservative treatment protocols.

### 3.4. Recovery of physical abilities at final follow-up

All patients successfully regained the ability to walk and perform daily activities without significant complications. The average time required for these functions to normalize was 7.3 ± 1.6 weeks, within a range of 6 to 9 weeks. Of the patients who had been previously engaged in sports activities (n = 13), only 3 continued to participate in sports preoperatively. Remarkably, 12 of these 13 patients resumed sports activity postoperatively. The time required for resumption ranged from 8 to 24 weeks, averaging 13.4 ± 1.7 weeks. This indicates a high rate of successful return to preoperative physical activity levels, particularly in the sports domain. The average time for complete recovery of normal activities (7.3 weeks) and the return to sports activities (13.4 weeks) offer insightful parameters for evaluating surgical outcomes. These metrics not only establish the efficiency of surgical intervention but also suggest that patients can expect a relatively quick return to both daily and recreational activities.

## 4. Discussion

Athletic trauma is one of the most frequent causes of damage to ankle cartilage. For instance, ankle injuries occur in one out of every 10,000 individuals daily. Non-traumatic OLT cases are relatively rare and can result from chronic ankle instability, talar osteonecrosis, hormonal and drug-related factors, and degenerative osteoarthritis.^[11,12]^ In our study involving 20 patients, 7 had direct cartilage surface injuries due to athletic trauma, while 13 had OCD resulting from post-traumatic conditions. The cartilage in the ankle joint is thinner and contains more proteoglycan and water than knee cartilage. This anatomical distinction explains why primary osteoarthritis is less common in the ankle than in other lower limb joints, making OLT more frequently associated with OCD.^[13,14]^ In our patient cohort, the OLT predominantly occurred in the talar dome. This observation can be attributed to biomechanical stress concentrated on the talar surface, which bears the weight of the entire body. Previous research has shown conflicting data regarding the prevalence of medial versus lateral OLT. In our study, 7 lesions were located laterally and 13 were located medially, possibly reflecting the anatomical and biomechanical characteristics of the ankle joint.

Various techniques, such as arthroscopic debridement, subchondral drilling, microfracture, and allogenic or autologous cartilage transplantation, are commonly employed to treat OLT, each with its advantages and disadvantages. The absence of vascular supply to the articular cartilage renders self-repair improbable. Traditional methods, such as arthroscopic debridement coupled with drilling or microfracture, aim to induce mesenchymal stem cells from the bone marrow into the lesion.^[15]^ However, these techniques have limitations, such as the formation of fibrocartilage, which lacks the durability of the native cartilage. Risk factors that adversely affect cartilage repair include the depth and size of the cartilage defect and the patient’s age. Our experience suggests that full-thickness cartilage injuries larger than 1.0 cm^2^ and in patients over 35 years of age are unlikely to achieve complete or partial coverage through drilling or microfracture. ACI is a new technology. ACI involves in vitro culturing of healthy cartilage tissue followed by implantation into the lesion. However, this method has several limitations, including low postoperative cell survival rates, phenotypic instability, and inadequate integration with surrounding tissue.^[16,17]^

Matrix-induced autologous chondrocyte implantation has emerged as a leading technology for cartilage repair. It improves cell survival rates and offers advantages, such as smaller surgical incisions and no size limitations for repair. However, the cost and lack of long-term outcome data have limited its widespread adoption. AOT is a second-generation cartilage repair technology that has gained widespread acceptance in Europe and America.^[7,18]^ This is gradually becoming a focus of domestic research. AOT provides a comprehensive matrix, viable chondrocytes, intact tidemark, and strong osseous support, making it a promising treatment option for OLT.

Our study provides a comprehensive analysis of AOT and other surgical techniques for treating OLT at Hepple stage IV and V. The significant improvement observed in VAS, AOFAS, and MOCART scores (*P* < .05) not only substantiates the effectiveness of the surgical interventions but also highlights their potential to significantly enhance patient outcomes in terms of pain reduction, functional recovery, and cartilage regeneration. Radiographic assessments provided substantial evidence of cartilage healing post-surgery, where 5 cases displayed hypertrophic cartilage fully covering the defect area, and 12 showed complete coverage with normal thickness. This suggests that the employed surgical techniques are not only effective in treating the surface level symptoms but are also instrumental in promoting deep tissue recovery. This capability to restore cartilage integrity is crucial, considering the chronic nature of OLT and the challenges associated with its long-term management. The stability of IKDC scores post-surgery (*P* > .05) across all participants is particularly noteworthy. It indicates that the surgical procedures utilized, including the harvesting of osteochondral grafts, do not adversely affect the donor sites, thereby addressing a common concern associated with autologous transplantation techniques. This aspect of the study is of significant clinical importance as it reassures patients and clinicians about the safety of the donor site, which can often be a deterrent to opting for such surgical solutions. The rapid recovery timeline, with patients returning to normal activities within an average of 7.3 weeks and resuming sports by 13.4 weeks, underscores the minimal disruptive nature of the surgery and its conducive role in quick patient turnaround. This is particularly beneficial in a sports medicine context, where athletes’ time away from training is critical.

Our study provides critical insights into the treatment of OLT at the more complex Hepple stages IV and V. These stages represent advanced cartilage damage, making effective treatment a significant challenge. By focusing on these stages, our findings make a substantial contribution to the existing literature, offering evidence that the surgical techniques employed can effectively manage even the most severe cases. Moreover, our comprehensive evaluation of pain, functional recovery, and radiological outcomes offers a holistic view of treatment efficacy. This not only sets a benchmark for future studies but also ensures a multi-dimensional approach to assessing surgical interventions. Additionally, the detailed documentation of surgical techniques and postoperative care enhances the practical application of these findings in clinical settings. By providing a replicable and detailed protocol, our study serves as a valuable guide for orthopedic surgeons, potentially improving clinical practices and patient outcomes in the treatment of complex OLT cases.

Although the results are promising, several limitations of this study warrant consideration. First, the sample size was relatively small, which limits the generalizability of our findings. However, we believe that our findings hold statistical significance and reliability, despite the relatively small sample size. The robustness of our statistical analysis compensates for this limitation and supports the dependability of our conclusions. Second, the follow-up duration varied among the participants, potentially affecting the longitudinal validity of the data. Third, we did not include a control group, which makes it difficult to isolate the impact of surgical intervention from other potential variables. Lastly, the study focuses on quantitative metrics and largely overlooks qualitative factors, such as patient satisfaction or quality of life, which are crucial in a comprehensive evaluation of surgical outcomes.

Future research on osteochondral lesions of the talus should extend follow-up beyond 1 year to assess long-term efficacy and complications, increase sample sizes and diversify patient demographics for broader applicability, and implement controlled, comparative studies to isolate treatment effects. Incorporating qualitative outcomes like patient satisfaction and standardizing surgical protocols will enhance outcome reliability and clinical integration. Additionally, economic analyses are crucial to evaluate the cost-effectiveness of surgical options, addressing healthcare resource utilization.

## 5. Conclusions

AOT is an efficacious and safe intervention for the treatment of patients with Hepple stage IV to V OLT, significantly improving ankle function, facilitating cartilage regeneration, and enabling a quick return to daily and sports activities without compromising donor-site integrity. Although promising, further research with larger sample sizes and control groups is essential to validate these findings.

## Author contributions

**Formal analysis:** Lin Liu, Ming Li.

**Writing – original draft:** Lin Liu.

**Data curation:** Ming Li, Sheng Liu.

**Investigation:** Fei Huang.

**Methodology:** Fei Huang, Sheng Liu.

**Resources:** Pengwei Li.

**Software:** Pengwei Li.

**Supervision:** Heda Liu.

**Writing – review & editing:** Heda Liu.
